# Comparison of Mechanical and Surface Properties between Conventional and CAD/CAM Provisional Restorations

**DOI:** 10.1055/s-0044-1791965

**Published:** 2024-12-10

**Authors:** Napatsorn Wechkunanukul, Kornuma Klomjit, Thawanrat Kumtun, Pongsiri Jaikumpun, Santiphab Kengtanyakich, Awutsadaporn Katheng

**Affiliations:** 1 Faculty of Dentistry, Naresuan University, Phitsanulok, Thailand; 2 Department of Restorative Dentistry, Faculty of Dentistry, Naresuan University, Phitsanulok, Thailand

**Keywords:** CAD/CAM, dental prosthetics, mechanical strength, PMMA, provisional restorations

## Abstract

**Objective** This study compared the flexural strength, surface hardness, and surface roughness of conventional, milled, and three-dimensional (3D)-printed provisional restorations.

**Materials and Methods** Bar-shaped polymethyl methacrylate (PMMA) specimens (25 × 2 × 2 mm ^3^ ) and disc-shaped specimens (9 × 2 mm ^2^ ) were fabricated using three different techniques ( *n* = 10/group): conventional (SR Ivocron C&B, Ivoclar Vivadent, Schaan, Liechtenstein), milling (Aidite Temp PMMA Blocks, Aidite, Qinhuangdao, China), and 3D printing (Asiga DentaTOOTH, Asiga, Sydney, Australia). Flexural strength was evaluated using a universal testing machine until fracture occurred. Vickers hardness and surface roughness tests were performed on the disc-shaped specimens using a micro-Vickers hardness tester and atomic force microscopy, respectively.

**Statistical Analysis** Data were statistically analyzed using one-way ANOVA. The post hoc Tukey's honest significant difference was conducted to compare the differences value between groups ( *p* < 0.05).

**Results** The milled computer-aided design/computer-aided manufacturing (CAD/CAM) provisional restorative material exhibited a significantly higher flexural strength (125.16 ± 6.83 MPa) compared with both the traditional (109.74 ± 14.14 MPa) and 3D-printed (71.09 ± 9.09 MPa) materials ( *p* < 0.05). The conventional material had a higher Vickers hardness (19.27 ± 0.41 kgf/mm ^2^ ) compared with the milled (18.53 ± 0.32 kgf/mm ^2^ ) and 3D-printed (17.80 ± 1.85 kgf/mm ^2^ ) materials, though the difference was statistically significant only between the conventional and 3D-printed groups. The surface roughness of the milled CAD/CAM material (8.80 ± 2.70 nm) was significantly lower than that of the 3D-printed material (24.27 ± 9.82 nm) ( *p* < 0.05).

**Conclusion** The provisional restorations fabricated using milled PMMA technology provide adequate flexural strength, surface hardness, and low surface roughness, offering a viable alternative for creating provisional restorations.

## Introduction

A provisional restoration is a fixed, removable, or maxillofacial prosthesis designed to protect oral structures and enhance aesthetics, stabilization, and function. 1 It is used between tooth preparation and the placement of the definitive restoration. 2 Polymethyl methacrylate (PMMA) is widely used for the fabrication of provisional restorations. Heat-polymerized PMMA, commonly referred to as conventional acrylic resin, is favored because of its availability, cost-effectiveness, and relative ease of repair. 3 However, production of PMMA restoration may be difficult to control quality from the laboratory procedures, and durability of provisional acrylic resin restorations is influenced by several factors, including the type of material used, the method of fabrication, the patient's oral environment, and the load applied during function. 1 Moreover, the heat generated during PMMA polymerization results in soft tissue damage and malodor, resulting in patient discomfort. 4

Computer-aided design/computer-aided manufacturing (CAD/CAM) has significantly impacted restorative dentistry. 5 6 7 CAD/CAM prostheses can be fabricated using either additive manufacturing (AM) or subtractive manufacturing (SM). AM methods create three-dimensional (3D) objects in a layer-by-layer process that offers high-resolution printing of complex geometries while reducing material waste and time consumption. 8 AM techniques are classified into seven types, with vat photopolymerization being the most popular. This category includes stereolithography (SLA), digital light processing (DLP), and liquid crystal display (LCD) methods. These techniques use photopolymerization to build a 3D structure from a photopolymerizable liquid monomer. In contrast, SM uses a milling machine to cut prefabricated disks or block-shaped materials. 9 Although milling produces high-quality dentures because of the highly cross-linked polymerization, it generates significant waste and requires large quantities of raw material. 10

Flexural strength is a critical physical property of materials used in oral rehabilitation, as fractures in temporary dental restorations can cause patient discomfort and lead to increased costs and treatment time. 10 A higher flexural capacity is essential for the clinical function of interim prostheses. Hardness indicates a material's resistance to plastic deformation from abrasion forces. Lower hardness usually correlates with low abrasion resistance and susceptibility to scratches. Because of its low hardness, PMMA is prone to scratching, which can lead to the formation of microcracks. These microcracks can weaken dental prostheses and promote bacterial growth. 11 Surface roughness affects the longevity of dental prostheses, potentially causing minor tissue trauma and increasing microorganism entrapment. This can contribute both directly and indirectly to tissue damage and a higher incidence of oral diseases. 12

In terms of fabrication, the production of provisional restorations using CAD/CAM technology can reduce patient visits. Moreover, these methods are precisely reproducible since data from the library storage can be used, which is advantageous for patients undergoing full-mouth rehabilitation or for those requiring long-term use. 3 4 In the event that prostheses become damaged, this approach reduces the steps and duration of treatment, leading to cost-effective outcomes. 4

However, a literature search revealed a lack of evidence-based information comparing 3D-printed and milled PMMA with conventional PMMA provisional restoration materials, particularly regarding their mechanical and surface properties which affect the longevity of the restorations. The research question of this study is: Is there a difference in flexural strength, surface hardness, and surface roughness between conventional, milled, and 3D-printed provisional restorations? Therefore, this study aims to compare the flexural strength, surface hardness, and surface roughness of conventional, milled, and 3D-printed provisional restorations. To use digital CAD/CAM for provisional restorations is a suitable alternative to the conventional approach. The hypothesis was that the CAD/CAM technique has higher flexural strength, surface hardness, and surface roughness compared with conventional provisional restorations.

## Materials and Methods

### Study Design and Specimen Preparation

**Fig. 1:**
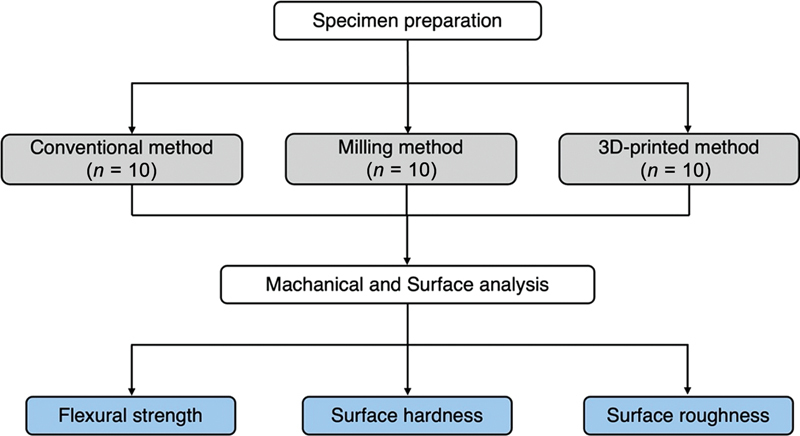
Flowchart of the experimental process for measuring the flexural strength, surface hardness, and surface roughness of specimens fabricated using different manufacturing methods.

The study design and workflow of the experimental process are illustrated in Fig. 1 . A total of 90 specimens were used in this study. The compositions of the materials used and their manufacturers are listed in Table 1 . The specimens for the flexural strength test were prepared as bar-shaped samples with dimensions of 25 mm × 2 mm × 2 mm, following the guidelines outlined in ISO 10477:2020. 13 For surface hardness and roughness testing, disc-shaped specimens with a diameter of 9 mm and a thickness of 2 mm were fabricated following the guidelines outlined in ISO 20795–1:2013. 14 Three different fabrication techniques for provisional restorations were utilized in this study: conventional heat-polymerization, milling, and 3D printing.

**Table 1:** Summary of materials used in the present study

Material	Manufacturer	Fabrication technique	Composition	Lot number
SR Ivocron C&B	Ivoclar Vivadent, Schaan, Liechtenstein	Conventional/heat cured	Powder: Methyl methacrylate, ethyl hexyl acrylate, N-octyl methacrylate Liquid: Methyl methacrylate, glycol dimethacrylate, dimethyl p-toluidine	YB61GR
Aidite Temp PMMA Blocks	Aidite, Qinhuangdao, China	Milling	Polymethyl methacrylate, methyl methacrylate, pigment	35293100
Asiga DentaTOOTH	Asiga, Sydney, Australia	3D printing	7,7,9(or 7,9,9)-trimethyl-4,13-dioxo-3,14-dioxa-5,12-diazahexadecane-1,16-diyl bismethacrylate, tetrahydrofurfuryl methacrylate, diphenyl (2,4,6-trimethylbenzoyl), phosphine oxide	M0/11068

Abbreviation: 3D, three dimensional.

Conventional Technique

Bar-shaped (25 × 2 × 2 mm ^3^ ) and disc-shaped (9 × 2 mm ^2^ ) wax samples were prepared. Paris plaster was poured into the lower body of the flask, and the wax samples were placed halfway into the plaster. Subsequently, more Paris plaster was poured into the upper body of the flask. Wax was removed using a boiling-out automat at 90°C for 10 minutes. The powder and liquid components of the heat-cured acrylic resin (SR Ivocron C&B, Ivoclar Vivadent, Schaan, Liechtenstein) were mixed according to the manufacturer's instructions and placed in a thermostat-controlled water bath at 70°C for 30 minutes, followed by immersion at 100°C for 80 minutes. 15 After deflasking, the samples were polished using 400, 800, 1000, and 1200-grit silicon carbide paper (Carbimet, Buehler, Illinois, United States), and finally with 0.05 μm aluminum oxide (Micropolish II, Buehler, Illinois, United States) using a polishing machine (Ecomet 30, Buehler, Illinois, United States).

Milling Technique

Identically sized and shaped specimens were designed using CAD software (3D Builder Version 18.0.1931.3, Microsoft Corporation, Washington, United States) and exported as Standard tessellation language (STL) files to CAM software for milling. The CAM software was used to instruct the milling machine to fabricate the samples. Prepolymerized acrylic resin blocks (Aidite Temp PMMA Blocks, Aidite, Qinhuangdao, China) were machined using a milling machine (DWX-52D, DGSHAPE Corporation, Hamamatsu, Japan) under dry conditions. 16 After milling, all specimens were polished using the same protocol as that used for the conventional group.

3D Printing Technique

The specimens were initially designed and exported as STL files using the CAD software. The specimens were then printed using a DLP 3D printer (ASIGA composer, Asiga, Sydney, Australia) with a resin color shade A2 (Asiga DentaTOOTH, Asiga, Sydney, Australia) and a layer thickness of 50 μm. The light source was a 405 nm UV LED with a laser power of 13.114 mW/cm ^2^ . After printing, the specimens were detached from the platform, washed with 99% isopropyl alcohol (KT Chemicals, Nishi, Osaka, Japan) for 10 minutes 17 using an ultrasonic cleaner (Sonorex super 10P, Bandelin, Vienna, Austria), and postprocessed in a UV oven (Hilite power 3D, Heraeus, Sydney, Australia) for 30 minutes at 40°C. 18 After printing, all specimens were polished using the same protocol as that used for the conventional group.

### Testing Protocol

Flexural Strength Test

For the three-point flexural strength test, bar-shaped samples ( *n* = 10/group) were conditioned at 37°C for 24 hours before testing. A universal testing machine (ElectroPuls E1000, Instron, Massachusetts, United States) was used. The specimens were placed horizontally on two round supports that were parallel to each other, with a span length of 20 mm. The specimen was supported at two points equidistant from the ends, and the loading point was positioned at the center of the span. A uniaxial load was applied to the center of the specimen with a 5 kN load cell and a crosshead speed of 5 mm/min until fracture occurred. The maximum force before fracture was recorded in Newtons (N). The flexural strength was calculated as follows:

Flexural strength = 3 *FL* /2 *bh* ^2^ ,

where *F* is the maximum force applied to the specimen (N); *L* is the distance between the two supports (mm); *b* is the specimen width (mm); and *h* is the specimen height (mm). 13 19

Surface Hardness Test

Surface hardness was assessed using a micro-Vickers hardness tester (Zhu-S, Indentec, West Midlands, United Kingdom) by applying an indentation with a load of 300 g for 15 seconds. 20 The strength was randomly applied to three areas of each disc-shaped specimen ( *n* = 10 per group, totaling 30 areas). Subsequently, the diagonals of the resulting square traces were measured using a microscope to evaluate the hardness.

Surface Roughness Test

To investigate the surface roughness (arithmetic mean height, Sa), three areas per specimen were randomly observed ( *n* = 10 per group, totaling 30 areas) using an atomic force microscope (AFM) 21 (Flex-Axiom, Nanosurf, Liestal, Switzerland) equipped with a scanner capable of a maximum range of 50 × , 100 × , and 5 mm along the *x* -, *y* -, and *z* -axes, respectively. Probes with nominal spring constants and resonance frequency values of 10 N/m and 250 kHz, respectively, were used for the observations.

### Statistical Analysis

A pilot study was conducted to calculate the appropriate sample size using the software (G*Power, Version 3.1.9.2, Kiel University, Kiel, Schleswig-Holstein, Germany). A sample size of 10 per condition was required to obtain an effect size *f* of 0.45 and 80% power at 5% α error. Statistical analysis was conducted using a statistical software program (IBM SPSS Statistics v24.0, IBM Corp., New York, United States). The Shapiro–Wilk test was used to assess the normality of data distribution, while Levene's test was used to examine the homogeneity of variances. As the data were normally distributed, a one-way ANOVA test, followed by Tukey's honest significant difference test, was conducted to test for variance, with a significance level set at 0.05.

## Results

### Flexural Strength

The manufacturing method significantly affects the flexural strength, surface hardness, and surface roughness of provisional restorations. The average flexural strength ranged from 71.09 to 125.16 MPa ( Table 2 ). The highest flexural strength was observed in specimens produced by the milling method (125.16 ± 6.83 MPa), significantly higher than those produced by the conventional (109.74 ± 14.14 MPa) and 3D printing methods (71.09 ± 9.09 MPa; *p* < 0.05). The 3D printing method showed the lowest flexural strength, with significant differences found among all methods ( *p* < 0.05; Table 2 ).

**Table 2:** Mean and standard deviation of the flexural strength, surface hardness, and surface roughness for each group ( *n* = 10)

Group	Flexural strength (MPa)	Surface hardness (kgf/mm ^2^ )	Surface roughness (nm)
Conventional	109.74 ± 14.14 ^b^	19.27 ± 0.41 a	12.27 ± 2.66 ^b^
Milling	125.16 ± 6.83 a	18.53 ± 0.32 a,b	8.80 ± 2.70 ^b^
3D printing	71.09 ± 9.09 ^c^	17.80 ± 1.85 ^b^	24.27 ± 9.82 a

Abbreviation: 3D, three dimensional.

a,b Indicate statistically significant differences between groups (rows) ( *p* < 0.05).

### Surface Hardness

The surface hardness values of the groups are listed in Table 2 . The highest surface hardness value was observed in the specimens produced via the conventional method (19.27 ± 0.41 kgf/mm ^2^ ), which was significantly different from that of the 3D printing method (17.80 ± 1.85 kgf/mm ^2^ ) *(p* < 0.05), but comparable to that of the milling method (18.53 ± 0.32 kgf/mm ^2^ ).

### Surface Roughness

The average values for surface roughness evaluation are listed in Table 2 . The milling group exhibited the lowest mean roughness (8.80 ± 2.70 nm), which was comparable to the conventional group (12.27 ± 2.66 nm). The highest surface roughness was observed for the 3D printing method, which significant differences noted among the various methods *(p* < 0.05).

## Discussion

CAD/CAM-processed prostheses have advanced significantly due to their convenience for both patients and dentists. 22 Even when using the same material, different manufacturing methods can alter the properties of PMMA. Therefore, this study compared the flexural strength, surface roughness, and surface hardness of 3D-printed and milled denture teeth with those of conventional denture teeth made of PMMA resin. These three critical properties influence the longevity of provisional restorations, leading to successful treatment outcomes. Results based on this study indicate that CAD/CAM manufacturing methods significantly affect the flexural strength, surface hardness, and surface roughness of provisional restorations, leading to the rejection of the hypothesis.

Higher flexural strength enhances a material's resistance to breaking, which is crucial for the clinical effectiveness of interim prostheses. The results demonstrated that the milling method produced the highest flexural strength, significantly different from those produced using the conventional and 3D printing methods. Previous studies have consistently shown that the flexural strength of milled PMMA specimens was superior to that of 3D-printed PMMA. 23 24 25 This is due to the optimal curing conditions and highly cross-linked methacrylic acid ester-based polymer in milled PMMA, which increases its mechanical properties. 26 On the other hand, the 3D printing method showed the lowest flexural strength, likely due to challenges in terms of building, postcuring, and minimal layer thickness, which can cause specimen shrinkage and modification during data conversion and STL file manipulation. 26 Conventional PMMA resins are monofunctional, low-molecular-weight, linear molecules, contributing to lower strength and rigidity. The absence of pressure during polymerization can trap air bubbles, further reducing strength. Residual monomer content also affects strength, polymerization shrinkage, and other material properties. 27 Therefore, the milled PMMA specimen exhibited the highest flexural strength, followed by the conventional and 3D-printed PMMA specimens.

Regarding the surface hardness, an indicator of a material's resistance to permanent surface indentation or penetration, was compared across different fabrication methods. A higher surface hardness generally indicates reduced surface alteration and bacterial growth. 11 28 The conventional method exhibited the highest surface hardness, significantly surpassing that of the 3D printing method, although it was not notably different from the milling method. In contrast, the 3D printing group showed the lowest surface hardness, likely due to the high residual monomer content and porosity of 3D-printed PMMA as reported by Souza et al. 25 Surface hardness can be related to material density, with denser materials being more resistant to wear and surface deterioration. PMMA block processing produces polymers with high density, resulting in high surface hardness. 26 In contrast, Digholkar et al 27 found that conventional PMMA had a significantly higher surface hardness than milled PMMA. Farina et al 23 reported that the homogenous heating of PMMA yielded higher monomer conversion, lowered the plasticizing effect of residual monomers, and increased surface hardness, supporting the superior behavior of conventional PMMA. The differences in results between studies 27 may arise from the different methods used, the sample size, dimensions, and the types and manufacturers of PMMA used.

Surface roughness, a measure of the height variation between points on a surface relative to its arithmetic mean, is an important factor in evaluating the quality of dental materials. In this study, the results demonstrated that the surface roughness of the PMMA specimens fabricated using the 3D printing method exhibited the highest values owing to their greater porosity. This indicates a statistically significant difference compared with the conventional and milling methods, which showed lower roughness due to their lower residual monomer content. A higher roughness value leads to greater tissue damage and increased bacterial accumulation, increasing the risk of oral diseases.

**Fig. 2:**
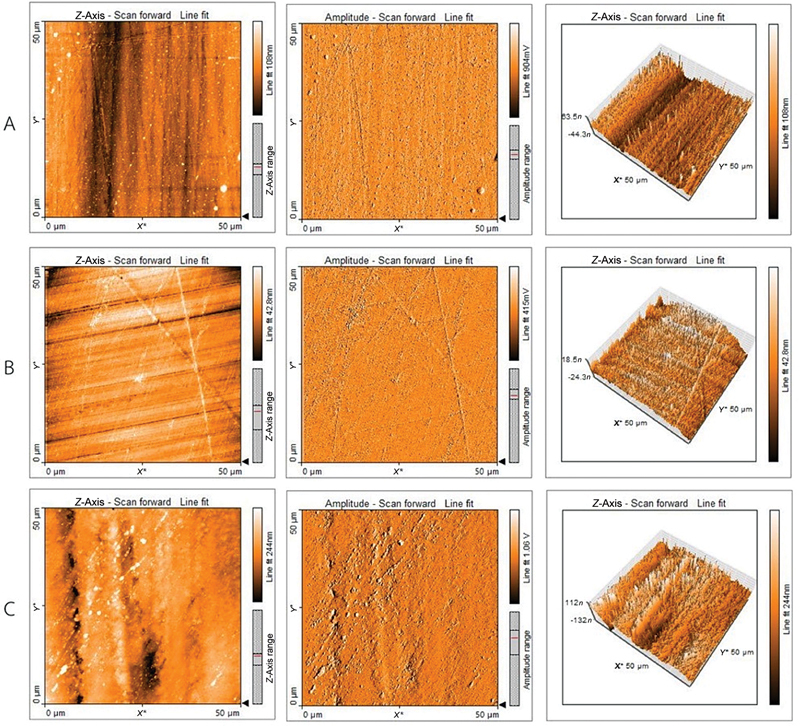
Representative surface roughness images of scanned areas for the conventional group (A), milled group (B), and 3D-printed group (C). 3D, three dimensional.

However, a limitation of this study is the use of AFM to measure surface roughness in Sa, a metric that differs from the Ra measurements used in previous studies and the clinical threshold for plaque accumulation (Ra = 0.2 μm). 28 29 The AFM images show exaggerated Z range features, indicating that the surface characteristics of the 3D-printed specimens are more pronounced than those of the conventional and milled specimens ( Fig. 2 ). This contradicts a previous study, 28 wherein no significant differences were found in the surface roughness values between the milling and 3D printing methods. However, that study used a profilometer to measure surface roughness, whereas our study measured it using AFM. Moreover, different polishing methods can yield different surface roughness results. Further studies should investigate additional properties of various manufacturing methods, such as optical characteristics, and assess the cyclic loading resistance of these materials to better understand their suitability for clinical use.

## Conclusion

Based on the findings of this *in vitro* study, we conclude that different manufacturing methods can alter the flexural strength, surface hardness, and surface roughness of PMMA materials. The provisional restorations fabricated using milled PMMA technology provide adequate flexural strength, surface hardness and low surface roughness, making them suitable for dental applications.
